# High tumor cell expression of microRNA-21 in node positive non-small cell lung cancer predicts a favorable clinical outcome

**DOI:** 10.1186/1472-6890-14-9

**Published:** 2014-02-13

**Authors:** Helge Stenvold, Tom Donnem, Sigve Andersen, Samer Al-Saad, Andrej Valkov, Mona Irene Pedersen, Lill-Tove Busund, Roy M Bremnes

**Affiliations:** 1 Institute of Clinical Medicine, University of Tromso, Tromso, Norway; 2 Department of Oncology, University Hospital of North Norway, Tromso 9038, Norway; 3 Institute of Medical Biology, University of Tromso, Tromso, Norway; 4 Dept of Clinical Pathology, University Hospital of North Norway, Tromso, Norway

**Keywords:** NSCLC, Stage I-IIIA, Survival, Prognostic impact, miR-21, miRNA

## Abstract

**Background:**

MicroRNA (miR)-21 has been revealed as an oncogene in cancer development, and is one of the miRNAs closely connected to angiogenesis. We aimed to explore the impact of miR-21 expression in both tumor and stromal compartments of non-small cell lung cancer (NSCLC), and correlations between miR-21 and angiogenic protein markers.

**Methods:**

From 335 unselected stage I to IIIA NSCLC carcinomas, duplicate tumor and tumor-associated stromal cores were collected in tissue microarrays (TMAs). *In situ* hybridization (ISH) was used to detect the expression of miR-21 separately in tumor cells and stromal cells of the tumor, and immunohistochemistry (IHC) was used to detect the expression of the protein markers protein kinase B (Akt), phosphatidylinositol-3-kinase (PI3K), hypoxia induced factor 1 (HIF1α) and vascular endothelial growth factor-A (VEGF-A).

**Results:**

In univariate analyses, high tumor cell expression of miR-21 in patients with lymph node metastasis was a positive prognostic factor (P = 0.024). High stromal miR-21 expression had a negative prognostic impact (P = 0.022). In the multivariate analysis, low tumor mir-21 expression in node positive patients was an independent adverse prognostic factor (HR 2.03, CI 95% 1.09-3.78, P = 0.027).

**Conclusions:**

In patients with lymph node metastasis, miR-21 expression in tumor cells is an independent positive prognostic factor. High stromal miR-21 expression is a negative prognostic factor.

## Background

Lung cancer is the leading cause of cancer-related deaths. NSCLC accounts for 80-85% of all lung cancers. New treatment strategies have so far had limited effect on lung cancer mortality [1]. Hence, research to identify new possible treatment targets is pivotal.

MicroRNAs (miRNAs) are small (19-22 nucleotides) non-coding RNAs. They play an important role in different cellular processes, such as regulation of proliferation, differentiation, apoptosis, development, metabolism, stress response and immunity [2,3]. It is assumed that approximately 30% of the genes are regulated by miRNAs [3]. The accruing prognostic data on miRNAs has made them interesting as potential therapeutic targets. Novel agents have not yet reached clinical trials, but there is considerable research going on in this field [4].

miR-21 is one of the most thoroughly studied miRNAs. Studies have revealed miR-21 as an oncogene [5], and in a recent meta-analysis miR-21 appeared as a negative prognostic factor [2]. It is also one of the miRNAs closely connected to angiogenesis [6,7]. In a recent study [8], we screened tissues from 10 worst and 10 best prognosis NSCLC cases as well as 10 controls for the expression of several angiogenesis-related miRNAs. miR-21 was significantly up-regulated in tumor versus normal tissue, and was among the miRNAs with the largest expression difference between tumor tissue and normal samples. Though recent studies on its influence on angiogenesis suggest both pro- and antiangiogenic properties [9,10], the involved mechanisms remains to be further investigated.

In our screening study [8] there was a four-fold change in tumor when compared to normal tissue when quantified by microarray hybridization and validated by real-time qPCR, but there was no significant difference between expressions in poor versus good prognostic cases. In fact, previously published results on the prognostic impact of miR-21 have been conflicting [11-15]. In cancer, the tumor stroma is, in addition to tumor cells, an important player in cancer development. Accordingly, miRNAs can be expressed differentially in tumor cells than in the surrounding stroma, and one speculate if its impact on prognosis could be different in the two compartments [16,17]. To further explore the prognostic impact of miR-21 in NSCLC we used ISH to facilitate, for the first time, evaluation of specific miR-21 expression in tumor cells and tumorsurrounding stromal cells, respectively.

In this study we aimed to investigate the prognostic impact of miR-21 in a large unselected NSCLC population. Since the impact of various angiogenic protein markers have been investigated in this cohort [18-20], we have also assessed the association between miR-21 and angiogenic markers.

## Methods

### Patients and clinical samples

Between 1990 and 2004, 371 patients with pathological stage I to IIIA non-small cell lung cancer were diagnosed at the University Hospital of North Norway and Nordland Central Hospital. Resected tissues from the primary tumors in these patients were used in our retrospective study. Out of 371 patients, 36 were excluded from the study due to radiotherapy or chemotherapy prior to surgery (n = 10), other malignancy within 5 years before NSCLC diagnosis (n = 13) or inadequate paraffin-embedded fixed tissue blocks (n = 13). Adjuvant chemotherapy was not introduced in Norway during this period (1990 – 2004). Thus, 335 patients with complete demographic and clinicopathological data were eligible for this study.

This report includes follow-up data as of January 10, 2011. The median follow-up time of survivors was 105 months (range 73-234). Formalin-fixed, paraffin-embedded tumor specimens were obtained from the archives of the Departments of Clinical Pathology at the University Hospital of North Norway and Nordland Central Hospital. The pathological data were revised according to the 7th edition of UICC TNM classification of lung cancer [21]. The National Data Inspection Board and the Regional Committee for Research ethics approved this study.

### Microarray construction

We used a 0.6 mm-diameter stylet to sample two cores with neoplastic tissue and two cores with tumor stroma from different areas of the primary tumors from each patient. The tumor stroma consists of the non-malignant cells of the tumor; activated fibroblasts, specialized mesenchymal cell types, innate and adaptive immune cells and the vasculature with endothelial cells and pericytes, as well as the extracellular matrix (ECM). Normal lung tissue localized distant from the tumor and lung tissue sample from 20 patients without cancer diagnosis were used as controls. The TMAs were assembled using a tissue-arraying instrument (Beecher Instruments, Silver Springs, MD, US). Eight tissue microarray blocks were made to include all the tissue samples. Multiple 4-μm-sections were cut with a Micron microtome (HM355S) and stained by specific antibodies for immunohistochemical analyses. The detailed methodology has been previously reported [20].

### In situ hybridization (ISH)

*In situ* hybridization was performed following the protocol developed by Exiqon, Vedbaek, Denmark [22]. Digoxigenin (DIG) labeled locked nucleic acid (LNA) modified probes from Exiqon for miR-21 (hsa-miR-21), positive control (U6, hsa/mmu/rno) and negative control (scramble-miR) from Kit 2, miR-21, (90002, Exiqon) were used in this study. Some adjustments were done to get a specific and sensitive detection of miRNA in our sections from formalin-fixed paraffin-embedded (FFPE) TMA blocks.

We placed 4 μm sections of the TMA blocks in a heater at 59°C over night to attach cores to Super Frost Plus slides. Sections were deparaffinised with xylene (3 × 5 min.) and then rehydrated with ethanol solutions (99.9% - 96% - 70%) ending up in PBS, pH 7.4. Proteinase-K (20 μg/ml) (Exiqon, Vedbaek, Denmark) treatment was done in PK-buffer (5 mM Tris.HCl, pH 7.5, 1 mM EDTA, 1 mM NaCl, autoclaved) at 37°C for 20 min in a HYBrite automated hybridizer (Abbot laboratories, IL, US). After a PBS wash the sections were dehydrated through increasing gradient of ethanol solutions and air-dried. The LNA-probes were denatured by heating to 90°C for 4 min. Hybridization of the LNA-probe miR-21 (50 nM) and scramble miR (50 nM) control was carried out in the HYBrite automated hybridizer at 50°C for 60 min. The positive control U6 (1 nM) was hybridized at 55°C for 60 min. Stringent washes was performed in pre-heated SSC buffers, 1 × 5 min in 5× SSC and 2 × 5 min in 1× SSC and 0,2× SSC. Sections were blocked against unspecific binding in blocking solution from DIG wash and Block Buffer set (Roche, Mannheim, Germany) for 15 min at room temperature (RT). Alkaline phosphatase (AP)-conjugated anti-DIG (Roche) 1:800 was incubated for 60 min at RT for immunologic detection. After PBS-T wash the substrate enzymatic reaction was carried out with NBT/BCIP (Roche) at 30°C in the hybridizer for 120 min. The reaction was stopped with a 2 × 5 min wash in KTBT buffer (50 mM Tris-Hcl, 150 mM NaCl, 10 mM KCl). Counter stain with nuclear fast red (WALDECK, ZE-012-250) was done at RT for 1 min and then rinsed in tap water, dehydrated through increasing gradient of ethanol solutions and mounted with Histokitt mounting medium (Assistant-Histokitt, 1025/250).

### Immunohistochemistry (IHC)

The detailed p-Akt Thr308 (rabbit monoclonal, clone 736E311, #4056, Cell Signaling Technology, 1:50), Akt2 (rabbit monoclonal, clone 54G8, #4057, Cell Signaling Technology, 1:18), Akt3 (rabbit polyclonal, #4059, Cell Signaling Technology, 1:8), PI3K (rabbit polyclonal, #4254, Cell Signaling Technology, 1:25), HIF1α (mouse monoclonal, NB100-131, Novus Biological, 1:35000), and VEGF-A (rabbit polyclonal, RB-1678, Neomarkers, 1:10) IHC procedures has been previously published [18-20]. For each antibody, including negative controls, the TMA staining were done in a single experiment.

### Scoring of ISH and IHC

**Figure 1:**
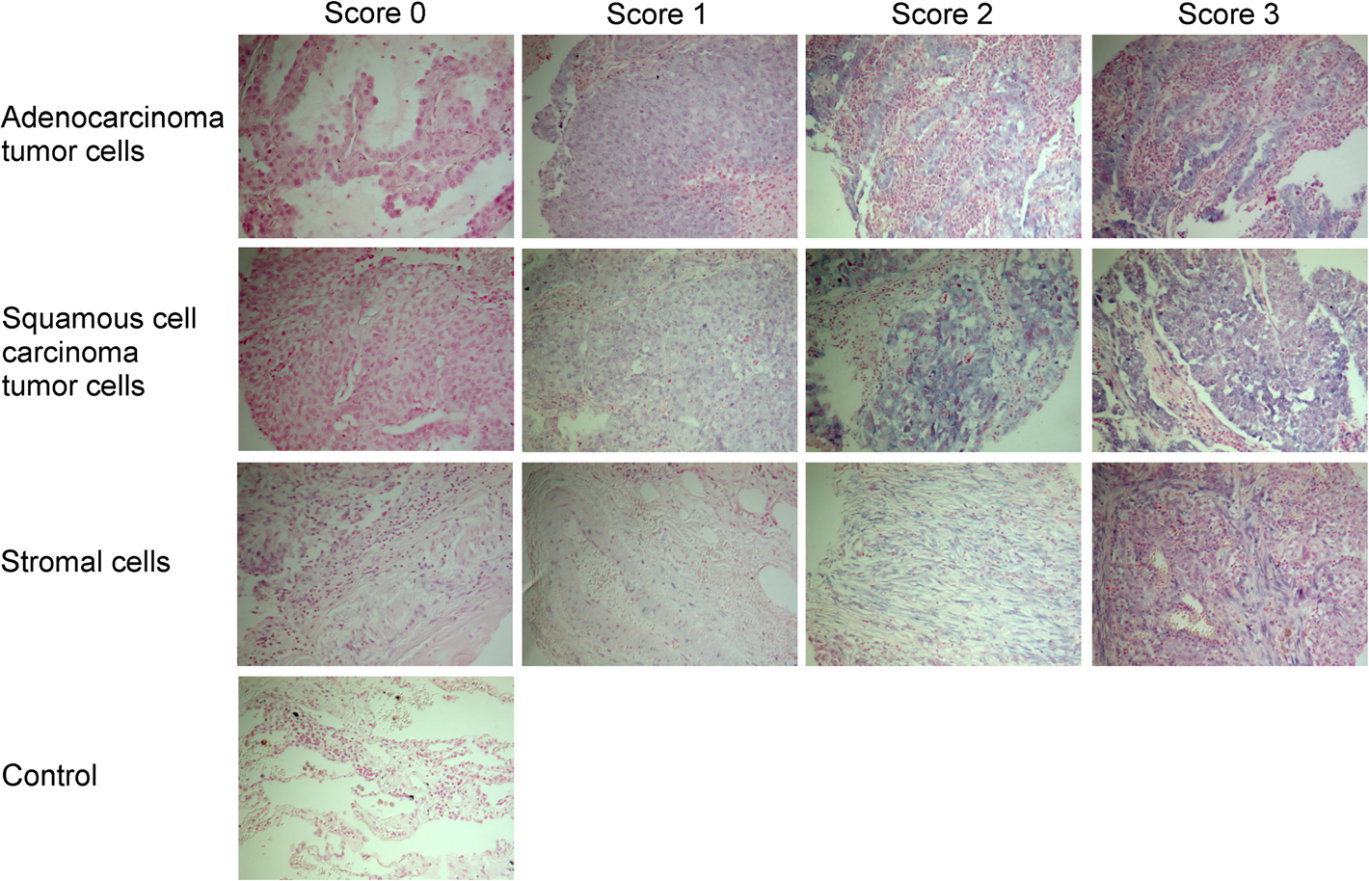
**In situ hybridization (ISH) analysis of non-small-cell lung cancer.** Scoring intensities based on blue cytoplasmatic staining graded from 0-3 differentiated in tumor cells and stroma of adenocarcinoma and squamous cell carcinoma are shown.

The ARIOL imaging system (Genetix, San Jose, CA) was used to scan the TMA slides of ISH staining. The slides were loaded in the automated loader (Applied Imaging SL 50) and specimens were scanned at low (1.25×) and high (20×) resolution using the Olympus BX 61 microscope with automated platform (Prior). Representative and viable tissue sections were scored manually and semiquantitatively for cytoplasmic staining on a computer screen. The dominating staining intensity in tumor cells was scored as: 0 = negative; 1 = weak; 2 = intermediate; 3 = strong (Figure 1). The tumor-related stroma was scored with one value from 0-3 based on both staining intensity and cell density. We summarized the scores from tumor cells and stroma to get a total score which may be comparable to findings in other studies using RT-qPCR, where it not is discriminated between tumor and stromal expression. All cores were anonymized and independently scored by 2 experienced pathologists (S.A.S. and A.V.). When assessing a variable for a given core, the observers were blinded to the scores of the other observer and to outcome. In case of disagreement (score discrepancy > 1), the slides was re-examined and a consensus was reached by the observers.

Mean score for each case was calculated from all 4 cores and both examiners. High expression of miR-21 in tumor cells was defined as a mean score ≥ 0.5. For stroma, high expression was defined as positive values (>0). For the angiogenic protein markers, the same cut-off values as previously published was used [18-20].

### Statistical methods

All statistical analyses were performed using the statistical package SPSS (Chicago, IL), version 19.0. The chi-square test and the Fisher exact test were used to examine the association between molecular marker expression and the clinicopathological markers. Correlations between markers were assessed using Spearman’s rank correlation. Plots of disease-specific survival (DSS) according to marker expression were drawn using Kaplan-Meier method, and statistical significance between survival curves was assessed by the log rank test. Variables of significant value from the univariate analyses were entered into multivariate analysis using the backward stepwise Cox regression analysis. A P < 0.05 was considered statistically significant.

## Results

### Patient characteristics

Demographic, clinical and histopathological variables are listed in Table 1. The median patient age was 67 (range 28-85) and the majority were male (76%). Most (95%) were current or previous smokers. The NSCLC tumors comprised 191 squamous cell carcinomas (SCCs), 113 adenocarcinomas (ACs) including 18 bronchioalveolar carcinomas (BACs) and 31 large-cell carcinomas (LCCs).

**Table 1:** Patient characteristics and their variables as predictors for disease-specific survival in 335 NSCLC patients (univariate analyses; log-rank test)

**Characteristics**	**Patients**		**Median survival month**	**5-year survival %**	**P**
	**n**	**(91)**			
**Age**					
≤65years	156	(47)	98	55	0.42
**>**65 years	179	(53)	NR	60	
**Sex**					
Female	82	(24)	190	64	0.22
Male	253	(76)	98	56	
**Smoking**					
Never	15	(5)	19	43	0.26
Current	215	(64)	NR	60	
Former	105	(31)	84	55	
**Performance status**					
PS 0	197	(59)	NR	63	**0.016**
PS 1	120	(36)	64	52	
PS 2	18	(5)	25	33	
**Weight loss**					
<10%	303	(90)	190	58	0.76
>10%	32	(10)	98	57	
**Histology**					
SCC	191	(57)	NR	66	**0.028**
Adenocarcinoma	113	(34)	54	46	
LCC	31	(9)	98	56	
**Differentiation**					
Poor	138	(41)	47	47	**<0.001**
Moderate	144	(43)	190	65	
Well	53	(16)	NR	68	
**Surgical procedure**					
Lobectomy + Wedge*	243	(73)	190	62	**0.007**
Pitunionectomy	92	(27)	37	47	
**Pathological stage**					
I	157	(47)	NR	61	**<0.001**
II	136	(40)	62	51	
IIIa	42	(13)	17	23	
**Tumor status**					
1	85	(25)	190	75	**<0.001**
2	188	(56)	84	57	
3	62	(19)	25	36	
**Nodal status**					
0	232	(69)	NR	67	**<0.001**
1	76	(23)	35	43	
2	27	(8)	18	18	
**Surgical margins**					
Free	3)7	(92)	190	59	0.37
Not free	28	(8)	47	48	
**Vascular infiltration**					
No	284	(85)	190	62	**0.001**
Yes	51	(15)	27	33	

*Wedge, n= 10.

Abbreviations: *NR* not reached, *PS* performance status, *SCC* squanos cell carcinoma, *LCC* large-cell carcinoma.

Statistically significant results in bold font.

### Expression of miR-21 and correlations

miR-21 was expressed in the cytoplasm of tumor cells. The staining was mainly diffuse and partly granular. In tumor stroma, inflammatory cells, pneumocytes, fibroblasts and endothelial cells also showed mainly diffuse cytoplasmic staining.

There were no significant correlations between miR-21 and the angiogenesis-related markers Akt, PI3K, HIF1α or VEGF-A. Neither were there any significant correlations when stratifying for nodal status (Table 2).

**Table 2:** Correlations between miR-21 expression and angiogenesis related markers

**Molecular marker**		**Akt**		**PI3K**		**HIF1α**		**VEGF-A**	
	**Compartment**	**Tumor**	**Stroma**	**Tumor**	**Stroma**	**Tumor**	**Stroma**	**Tumor**	**Stroma**
**A:** Correlations in all 335 patients									
**miR-21**	Tumor	P = 0.700		P = 0.488		P = 0.687		P = 0.751	
	Stroma		P = 0.217		P = 0.655		P = 0.251		P = 0.622
**B:** Correlations in 232 node-negative patients									
**miR-21**	Tumor	P = 0.736		P = 0.566		P = 0.473		P = 0.945	
	Stroma		P = 0.692		P = 473		P = 0.685		P = 0.272
**C:** Correlations in 103 node-positive patients									
**miR-21**	Tumor	P=0.735		P=0.701		P=0.751		P=0.685	
	Stroma		P=0.112		P=0.722		P=0.144		P=0.192

### Univariate analysis

As shown in Table 1, the clinicopathological variables performance status (P = 0.016), histology (P = 0.028), tumor differentiation (P < 0.001), surgical procedure (P = 0.007), pathological stage (P < 0.001), tumor status (P < 0.001), nodal status (P < 0.001) and vascular infiltration (P = 0.001) were significant prognostic indicators for DSS.

**Figure 2:**
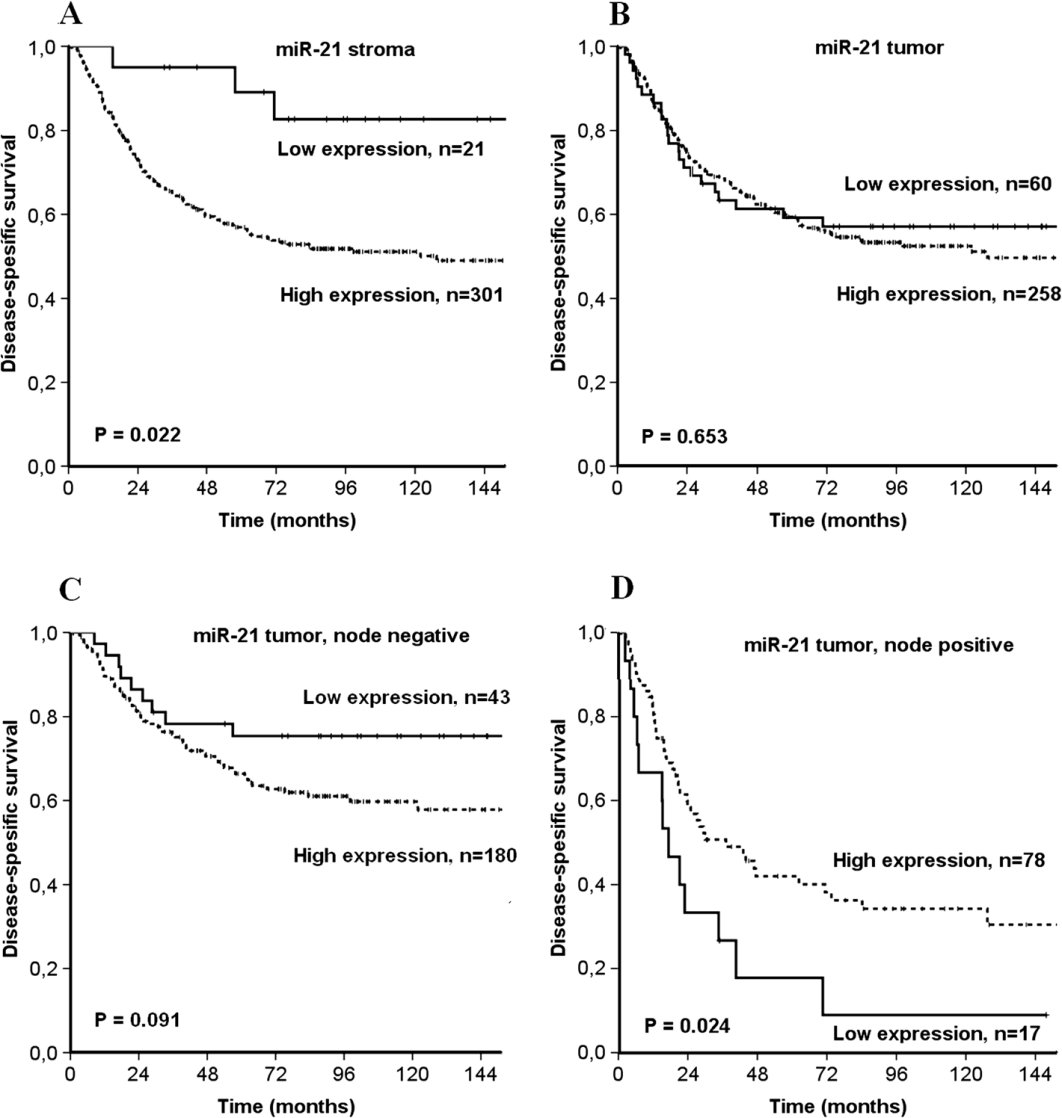
Disease-specific survival curves according to expression of A) miR-21 in stroma, B) miR-21 in tumor, C) miR-21 in tumor in node negative patients and D) miR-21 in tumor in node positive patients.

The survival analysis for miR-21 is presented in Table 3 and Figure 2. Expression of miR-21 in the total cohort based on both tumor and stromal cells had no significant prognostic impact.

**Table 3:** miR-21 in tumor cells and stroma as predictors for disease-specific survival in NSCLC patients (univariate analysis; log-rank test) and results of Cox regression analysis summarizing significant independent prognostic factors

**Characteristics**	**Pts (n)**	**Pts (%)**	**Median survival (months)**	**5-Year survival (%)**	**Univariate (P)**	**Multi-variate (P)**	**HR 95% CI**
**Total (n = 335)**					0.45	0.71	1.08
Low	220	66	190	61			0.73-1.60
High	98	29	98	55			
Missing	17	5					
**Tumor**^**1 **^**(n = 335)**					0.65	0.40	1.20
Low	60	18	NR	59			0.74-1.95
High	258	77	127	59			
Missing	17	5					
**N0 (n = 223)**					0.091	0.33	0.69
Low	43	19	NR	75			0.33-1.45
High	180	81	190	66			
**N+ (n = 95)**					**0.024**	**0.027**	**2.03**
Low	17	18	17	18			**1.09-3.78**
High	78	82	37	42			
**Stroma**^**2 **^**(n = 335)**					**0.022**	0.12	0.45
Low	21	6	189	89			0.16-1.24
High	301	90	127	57			
Missing	13	4					
**N0 (n = 225)**					**0.044**	0.061	0.32
Low	16	7	190	93			0.08-1.38
High	209	93	NR	66			
**N+ (n = 97)**					0.44	0.45	0.58
Low	5	5	71	75			0.14-2.37
High	92	95	27	36			

NR = not reached.

^1^Tumor cells.

^2^Cells of the peritumoral connective tissue (fibroblasts, mesenchymal cells, immune cells, endothelial cells, pericytes and extracellular matrix.

Statistically significant results in bold font.

The situation was the same when only tumor cells were assessed. In subgroup analyses of lymph node positive patients, however, a high tumor miR-21 expression was significantly associated with an improved prognosis when compared to low expression (P = 0.024). This was not observed in lymph node negative patients (P = 0.091).

In stroma of all patients, high miR-21 expression was a negative prognostic indicator (P = 0.022). This was also observed in the subgroup with node-negative disease, but not in node-positive patients.

### Multivariate analysis

In the multivariate analysis the following clinicopathological variables appeared as independent prognostic variables: performance status (P = 0.008), histology (P = 0.001), tumor differentiation (P = 0.007), tumor status (P = 0.007), nodal status (P = 0.022) and vascular infiltration (P = 0.004).

Results of the multivariate analyses for miR-21 expression are presented in Table 3. In analyses of the total material, all significant clinicopathological factors from the univariate analyses were included. For the N + subgroup, no relevant clinicopathological factors were significant in the univariate analysis. Tumor or stromal miR-21 expression in the total material had no independent prognostic impact. In node positive patients, however, low tumor miR-21 expression was an independent negative prognostic factor (HR 2.03, CI 95% 1.09-3.78, P = 0.027).

## Discussion

In a large unselected cohort, we have used high-throughput TMA-technique and *in situ* hybridization to evaluate the prognostic impact of miR-21 expression in tumor tissue and stroma of NSCLC. To our knowledge, we are the first to use ISH to study miR-21 expression and outcome in a large NSCLC cohort, facilitating analyses discriminating specifically between tumor cells and cells of the tumor stroma. We find high tumor miR-21 expression in node-positive patients to be an independent positive prognostic indicator. In univariate analyses, we find high stromal miR-21 expression to be a negative prognostic factor in the total material and in node-negative patients. There was no correlation between miR-21 and angiogenesis-related markers.

miRNAs have a large impact on gene regulation, and are considered major players in tumor development and metastasis [3]. These nucleotides act as both oncogenes and tumor suppressor genes, as they may be both up- and down-regulated in tumors. miR-21 is known to be abundantly expressed in a variety of cancers, and is in many tumor types associated with a reduced overall survival [2]. In a recent array screening study, including 20 NSCLC patients and 10 controls, we found miR-21 to be one of the most upregulated miRNAs in tumor tissue, when compared to normal tissue, by both microarray hybridization and quantitative real time polymerase chain reaction (qRT-PCR) technique [8].

In recent years, a few studies have explored the prognostic impact of miR-21 in NSCLC. Markou et al. studied 48 patients, where 67% where in stage I/II and 33% stage III/IV. They found miR-21 to be an independent negative prognostic factor for OS [13]. Gao et al. observed the same in 47 NSCLC samples. In their cohort, 47% were stage I, 25% stage II and 28% stage III, respectively [11]. In three cohorts from Maryland, US (64% stage I, 25% stage II, 11% stage III), Norway (57% stage I, 14% stage II, 29% stage III), and Japan (74% stage I, 26% stage II), Saito and colleagues showed miR-21 to be an independent negative prognostic factor. In the Norwegian and American material, overall survival was the endpoint, while in the Japanese cohort relapse free survival was [14]. Landi and colleagues used an oligo array with 440 human miRNAs to evaluate differences in miRNA expression depending on histology and clinical outcome in 290 NSCLC tissues, constituted by 40% stage I, 29% stage II, 26% stage III and 4% stage IV cancers. They found miR-21 to differentiate between adenocarcinoma and squamous cell carcinoma, but there was no difference in survival according to miR-21 expression rate [12]. In a large study on 639 patients (35% stage I, 23% stage II and 42% stage III), Voortman et al. observed no prognostic impact of miR-21 on NSCLC survival. There was a tendency towards a better prognosis for high miR-21 expression, but this finding was not significant (P = 0.06) [15].

There were some differences in methodology between these studies, as fresh frozen tissue was used in three [11,13,14] and paraffin-embedded material in two studies [12,15]. For quantification of miRNA, all except the Landi study used qRT-PCR. In the studies where miR-21 was associated with a worse prognosis, subgroup analyses were not performed. The Landi and Gao studies [11,12] were numerously too small and the Saito study consisting of three cohorts (89, 37 and 189 patients respectively) [14], was not suited for subgroup analyses.

Yang et al. performed a meta-analysis based on the studies mentioned above. They also included two other studies analyzing miR-21 in serum. The conclusion was that high miR-21 expression was significantly associated with poor survival [23].

When using the summarized score of tumor cell and stromal expression in the whole cohort, miR-21 was without any significant prognostic impact. In the stromal compartment, miR-21 expression was a negative prognosticator in univariate, but not in the multivariate analysis. In the studies mentioned above [11,13,14], except for the studies by Voortman [15] and Landi [12], miR-21 appears to be a negative prognostic factor in NSCLC, corroborating our stromal results. In contrast, we found miR-21 expression in tumor cells to be an independent positive prognostic factor in node positive lung cancer patients. One may speculate if the differences we observe between our results and some of the other studies are caused by the different methodologies used. As we use ISH-technique, miR-21 expression can be assessed separately in tumor and stromal cells. When using the qRT-PCR method without prior microdissection, it can not be differentiated between the tumor cells and the stromal compartment. Gregg and colleagues performed microdissection on a prostate cancer material to separate tumor and stromal cells, and showed a large difference regarding gene expression between the two compartments [24]. Our findings show that there is a prognostic difference in expression between the two compartments. Using qRT-PCR the contribution of miR-21 from the stromal compartment may override the contribution from tumor cells, especially at significant differences in expression. Consequently, the data will reflect the situation in stroma, and a divergent situation in the tumor cells will not be detected. The conclusion in the Yang meta-analysis reflects the findings in these studies, and does not take into account possible expression differences between the compartments.

The mechanistic functions of miR-21 are still being explored, but some functions have recently been suggested. In human umbilical vein endothelial cells (HUVECs), Sabatel and colleagues found miR-21 to be a potential inhibitor of angiogenesis via inhibition of RhoB, resulting in a reduction in endothelial proliferation, migration and vessel formation [10]. On the other hand, Liu et al. demonstrated miR-21 to induce angiogenesis in human prostate cancer cells through up-regulating HIF-1α and VEGF and through activating the Akt and ERK pathways [9]. Hence, miR-21 may have both pro- and anti-angiogenic functions. Angiogenesis is an important trait of cancer progression [25], and inhibiting angiogenesis may contribute to slow down cancer growth. In the light of these opposing findings and our data, we may speculate if miR-21 has opposite impacts in different stages of the disease. Could high miR-21 in early stages (node-negative) act pro-angiogenic and contribute to a faster progression, while in a node positive stage, it acts anti-angiogenic and protects against further progression?

We did not find any correlations between miR-21 and angiogenic markers of pathways earlier described for miR-21 and angiogenesis [9,10]. These pathways are possibly tissue and cell type specific. Studies exploring miR-21 as a modulator of angiogenesis have in some cases used endothelial cells in their models [10]. In our material, we have not specifically studied endothelial cells, so the connection between miR-21 and angiogenetic markers seen in endothelial cells will not necessarily be mirrored by our tissue samples as we assessed the sum off all stromal cell types.

## Conclusion

We found tumor cell miR-21 expression to be an independent positive prognostic factor in node-positive NSCLC. In the total material, stromal miR-21 expression was a negative prognostic factor in univariate analysis. We identified diverging impacts of miR-21 related to cell compartment and nodal status. These findings should be further explored, and may have implications for the future use of miR-21 in diagnostics and therapy.

## Competing interests

The authors declare that they have no competing interests.

## Authors’ contributions

HS participated in the design of the study, contributed to the clinical and demographic database, did the statistical analysis and drafted the manuscript. TD, SA and SAS contributed to the clinical and demographic database and SAS and MIP in making the TMAs. TD and SA contributed to the statistical analysis. SAS and AV scored the cores. MIP carried out the ISH. RB and LTB supervised and participated in the study design, result interpretation and writing. All authors read and approved the final manuscript.

## Pre-publication history

The pre-publication history for this paper can be accessed here:

http://www.biomedcentral.com/1472-6890/14/9/prepub
